# Sex differences in the prognostic significance of KRAS codons 12 and 13, and BRAF mutations in colorectal cancer: a cohort study

**DOI:** 10.1186/2042-6410-4-17

**Published:** 2013-09-10

**Authors:** Sakarias Wangefjord, Magnus Sundström, Nooreldin Zendehrokh, Kajsa Ericson Lindquist, Björn Nodin, Karin Jirström, Jakob Eberhard

**Affiliations:** 1 Department of Clinical Sciences, Division of Pathology, Lund University, Lund, Sweden; 2 Department of Immunology, Genetics and Pathology, Uppsala University, Uppsala, Sweden; 3 Department of Clinical Sciences, Division of Oncology, Lund University, Lund, Sweden

**Keywords:** KRAS mutation, Codon 12, Codon 13, BRAF mutation, Colorectal cancer, Sex, Prognosis

## Abstract

**Background:**

Activating KRAS and BRAF mutations predict unresponsiveness to EGFR-targeting therapies in colorectal cancer (CRC), but their prognostic value needs further validation. In this study, we investigated the impact of KRAS codons 12 and 13, and BRAF mutations on survival from CRC, overall and stratified by sex, in a large prospective cohort study.

**Methods:**

KRAS codons 12 and 13, and BRAF mutations were analysed by pyrosequencing of tumours from 525 and 524 incident CRC cases in The Malmö Diet and Cancer Study. Associations with cancer-specific survival (CSS) were explored by Cox proportional hazards regression, unadjusted and adjusted for age, TNM stage, differentiation grade, vascular invasion and microsatellite instability (MSI) status.

**Results:**

KRAS and BRAF mutations were mutually exclusive. KRAS mutations were found in 191/ 525 (36.4%) cases, 82.2% of these mutations were in codon 12, 17.3% were in codon 13, and 0.5% cases had mutations in both codons. BRAF mutations were found in 78/524 (14.9%) cases. Overall, mutation in KRAS codon 13, but not codon 12, was associated with a significantly reduced CSS in unadjusted, but not in adjusted analysis, and BRAF mutation did not significantly affect survival. However, in microsatellite stable (MSS), but not in MSI tumours, an adverse prognostic impact of BRAF mutation was observed in unadjusted, but not in adjusted analysis. While KRAS mutation status was not significantly associated with sex, BRAF mutations were more common in women. BRAF mutation was not prognostic in women; but in men, BRAF mutation was associated with a significantly reduced CSS in overall adjusted analysis (HR = 3.50; 95% CI = 1.41–8.70), but not in unadjusted analysis. In men with MSS tumours, BRAF mutation was an independent factor of poor prognosis (HR = 4.91; 95% CI = 1.99–12.12). KRAS codon 13 mutation was associated with a significantly reduced CSS in women, but not in men in unadjusted, but not in adjusted analysis.

**Conclusions:**

Results from this cohort study demonstrate sex-related differences in the prognostic value of BRAF mutations in colorectal cancer, being particularly evident in men. These findings are novel and merit further validation.

## Background

The successful treatment of colorectal cancer (CRC) relies on an early diagnosis, radical surgery and adequate adjuvant treatment. Presently, tumour stage at diagnosis is the most important prognostic factor. However, it is becoming increasingly clear that CRC is a highly heterogeneous disease with different genetic and molecular characteristics affecting intrinsic tumour aggressiveness, response to systemic treatment, and, hence, clinical outcome. Although many efforts have been made to find biomarkers to more accurately predict high-risk disease and to select patients for adjuvant treatment, none have proven good enough for use in clinical routine.

Activating mutations of proto-oncogenes KRAS and BRAF are common in CRC, causing unregulated downstream signalling in the Ras/Raf/MEK/MAP signal transduction pathway, in turn, affecting a variety of cellular responses such as proliferation, differentiation, migration, survival and apoptosis [1]. Approximately 40% of all colorectal tumours harbour a KRAS mutation, predominantly occurring in codon 12 or 13 [2]. While KRAS mutation has proven to be predictive of the resistance to epidermal growth factor receptor (EGFR)-inhibiting therapies [3,4], the prognostic value of KRAS mutation in CRC remains unclear. Numerous studies have investigated the relationship between KRAS mutation status and survival from CRC with divergent results; however, the majority of them are associating KRAS mutation with a poor prognosis [5-11]. Notably, while most studies did not consider specific mutations, accumulating evidence indicates that specific codon 12 and 13 mutations have a stronger impact on the functionality of the KRAS protein, and, hence, its impact on clinical outcome in CRC patients [5,12,13].

BRAF mutations have been reported in CRC at a frequency of 5%–18% with the vast majority being a V600E substitution [14]. BRAF mutation has also been linked to an impaired prognosis in CRC [9,15,16] and unresponsiveness to anti-EGFR drugs [17-19]. BRAF and KRAS mutations are, with rare exceptions, mutually exclusive [20].

The prognostic value of clinicopathological factors [21,22] and investigative biomarkers [23] may well differ in men and women, but to our best knowledge, no previous studies have investigated sex-related differences in the prognostic impact of KRAS and BRAF mutation in CRC. In the present study, we examined the associations of specific KRAS and BRAF mutations with clinicopathological and tumour biological characteristics, and survival, in 525 incident cases of colorectal cancer from a prospective population-based cohort study.

## Methods

### Study population

Until the end of follow-up in 31 December 2008, 626 incident cases of CRC had been registered in the prospective population-based cohort from the Malmö Diet and Cancer Study (MDCS) [22,24]. Patient and tumour characteristics of the cohort have been described in detail previously [23,25-27]. Ethical permission was obtained from the Ethics Committee at Lund University. Tissue microarrays have been constructed from 557 cases as previously described [23,25]. Immunohistochemical analysis of mismatch repair proteins MLH1, PMS2, MSH2 and MSH6 for the assessment of microsatellite instability (MSI) status has been described in [26], analysis of beta-catenin overexpression in [27], of cyclin D1 in [23], and p21, p27 and p53 in [28].

### Analysis of KRAS and BRAF mutation status

The PyroMark Q24 system (Qiagen GmbH, Hilden, Germany) was used for pyrosequencing analysis of KRAS and BRAF mutations in DNA from 1 mm formalin-fixed, paraffin-embedded tumour tissue cores taken from areas with >90% tumour cells. In brief, genomic DNA was extracted from tumour tissue using QIAamp MinElute spin columns (Qiagen) and DNA regions of interest were PCR-amplified (Veriti 96-Well Fast Thermal Cycler, Applied Biosystems Inc., Foster City, CA, USA). KRAS codons 12 and 13 were analysed using Therascreen KRAS Pyro Kit (Qiagen). Analysis of BRAF mutation hotspots in codons 600 and 601 was performed using previously published PCR primers [29] and a novel BRAF sequencing primer (5′-TGATTTTGGTCTAGCTACA-3′) which was designed using the PyroMark Assay Design 2.0 software (Qiagen). All samples with a potential low-level mutation were reanalysed.

### Statistical analysis

Associations between KRAS and BRAF mutation status and clinicopathological factors were explored by Pearson's Chi-square test. Kaplan-Meier analysis and log rank test were performed to illustrate the differences in cancer-specific survival (CSS). Cox proportional hazards regression was used for estimation of hazard ratio (HR) for death from CRC. A backward conditional method was used for variable selection in the multivariable model including age, gender, T stage, N stage, M stage, differentiation grade, vascular invasion, MSI status, and KRAS and BRAF mutation status. The interaction between investigative factors and sex was explored by a Cox model including the interaction variable. All survival analyses were repeated with overall mortality as endpoint and all tests were two-sided. A *p* value of 0.05 was considered significant. All statistical analyses were performed using IBM SPSS Statistics version 20.0.

## Results

### Distribution of KRAS and BRAF mutations

KRAS and BRAF mutations were successfully evaluated in 525 and 524 cases, respectively. The distribution of specific KRAS mutations is shown in Table 1. A total number of 334 (63.7%) tumours were KRAS wild-type and 191 (36.4%) were KRAS-mutated. Specifically, 156 (29.8%) cases harboured a KRAS codon 12 mutation, 34 (6.5%) a KRAS codon 13 mutation and 1 case (0.2%) had dual codons 12 and 13 mutations. The distribution of specific KRAS mutations did not differ between sexes (data not shown). KRAS and BRAF mutations were mutually exclusive. Further, 446 (85.1%) of the tumours were BRAF wild-type, 76 (14.5%) were BRAF V600E-mutated and 2 (0.4%) were BRAF K601E-mutated with a total of 78 (14.9%) cases harbouring a BRAF mutation.

**Table 1:** Distribution of KRAS mutations in 191 cases

**Codon**	**Sequence (amino acid)**	**Number (%)**
12	CGT (Arg)	2 (1.0)
	GAT (Asp)	57 (29.8)
	GTT (Val)	60 (31.4)
	TGT (Cys)	15 (7.9)
	AGT (Ser)	13 (6.8)
	GCT (Ala)	10 (5.2)
13	GAC (Asp)	33 (17.3)
	GTC (Val)	1 (0.5)

### Correlations of KRAS and BRAF mutations with clinicopathological and tumour biological parameters

As shown in Table 2, there was a significant association between KRAS wild-type tumours and MSI. Further, KRAS codon 13 mutation correlated with metastatic disease (M1) and p27 negativity. Notably, when KRAS codon 12-mutated tumours were compared with tumours being either KRAS wild-type or codon 13-mutated, there was a significantly higher proportion of mucinous tumours in the former category (*p* = 0.032 and *p* = 0.024).

**Table 2:** Associations of KRAS codons 12 and 13, and BRAF mutation status with clinicopathological and molecular characteristics

	**KRAS wild-type**	**Codon 12-mutated**	**Codon 13-mutated**	***p *value**	**BRAF wild-type**	**BRAF-mutated**	***p *value**
	**334 (63.7)**^**a**^	**156 (29.8)**^**a**^	**34 (6.5)**^**a**^		**446 (85.1)**^**a**^	**78 (14.9)**^**a**^	
Age							
Mean, median	70.5, 71.4	70.9, 71.4	68.5, 69.6	0.297^b^	70.2, 70.8	72.0, 73.1	0.017^b^
Range	51.5–85.6	50.9–84.0	49.8–83.7		49.8–86.6	51.5–84.3	
Sex							
Female	177 (53.0)	83 (53.2)	18 (52.9)	0.999	227 (50.9)	50 (64.1)	0.031
Male	157 (47.0)	73 (46.8)	16 (47.1)		219 (49.1)	28 (35.9)	
Tumour location							
Proximal	101 (30.4)	54 (34.6)	10 (29.4)	0.058	106 (23.8)	59 (77.6)	<0.001
Transverse	18 (5.4)	0 (0)	3 (8.8)		11 (2.5)	10 (13.2)	
Descending	15 (4.5)	12 (7.7)	1 (2.9)		24 (5.4)	4 (5.3)	
Sigmoid	66 (19.9)	38 (24.4)	7 (20.6)		110 (24.7)	1 (1.3)	
Rectum	132 (39.8)	52 (33.3)	13 (38.2)		195 (43.7)	2 (2.6)	
Missing	2	-	-		0	2	
T stage							
1	32 (10.0)	9 (6.0)	4 (13.3)	0.291	42 (9.9)	3 (4.1)	0.001
2	38 (11.9)	18 (11.9)	2 (6.7)		52 (12.2)	6 (8.1)	
3	201 (63.0)	104 (68.9)	16 (53.3)		278 (65.3)	43 (58.1)	
4	48 (15.0)	20 (13.2)	8 (26.7)		54 (12.7)	22 (29.7)	
Missing	15	5	4		20	4	
N stage							
0	180 (59.6)	84 (57.9)	15 (51.7)	0.710	241 (59.7)	37 (51.4)	0.353
1	69 (22.8)	39 (26.9)	7 (24.1)		97 (24.0)	19 (26.4)	
2	53 (17.5)	22 (15.2)	7 (24.1)		66 (16.3)	16 (22.2)	
Missing	32	12	5		42	6	
M stage							
0	275 (84.1)	128 (82.6)	22 (64.7)	0.018	366 (83.0)	59 (78.7)	0.363
1	52 (15.9)	27 (17.4)	12 (35.3)		75 (17.0)	16 (21.3)	
Missing	17	1	-		5	3	
Differentiation grade							
High	20 (6.2)	11 (7.1)	0 (0)	0.088	25 (5.7)	5 (6.8)	<0.001
Intermediate	222 (68.7)	121 (77.6)	26 (76.5)		340 (77.4)	30 (40.5)	
Low	81 (25.1)	24 (15.4)	8 (23.5)		74 (16.9)	39 (52.7)	
Missing	11	-	-		7	4	
Vascular invasion							
No	95 (51.1)	45 (47.4)	7 ( 38.9)	0.561	125 (49.4)	21 (46.7)	0.735
Yes	91 (48.9)	50 (52.6)	11 (61.1)		128 (50.6)	24 (53.3)	
Missing	148	61	16		193	33	
Tumour type							
Non-mucinous	269 (82.0)	114 (73.5)	28 (84.8)	0.073	360 (81.8)	50 (65.8)	0.001
Mucinous	59 (18.0)	41 (26.5)	5 (15.2)		80 (18.2)	26 (34.2)	
Missing	6	1	1		6	2	
MSI screening status							
MSS	245 (79.3)	137 (96.5)	31 (93.9)	<0.001	384 (93.2)	29 (40.3)	<0.001
MSI	64 (20.7)	5 (3.5)	2 (6.1)		28 (6.8)	43 (59.7)	
Missing	25	14	1		24	6	
Beta-catenin grades							
0–1	94 (29.7)	37 (25.0)	13 (38.2)	0.470	99 (23.4)	45 (59.2)	<0.001
2–3	108 (34.1)	48 (32.4)	9 (26.5)		138 (32.6)	26 (34.2)	
4–5	115 (36.3)	63 (42.6)	12 (35.3)		186 (44.0)	5 (6.6)	
Missing	17	8	-		23	2	
p53 status							
Negative	169 (53.1)	77 (51.3)	12 (37.5)	0.240	202 (47.6)	57 (75.0)	<0.001
Positive	149 (46.9)	73 (48.7)	20 (62.5)		222 (52.4)	19 (25.0)	
Missing	16	6	2		22	2	
p21 Expression							
Negative	43 (13.7)	22 (14.9)	5 (15.2)	0.931	63 (15.0)	7 (9.2)	0.180
Positive	271 (86.3)	126 (85.1)	28 (84.8)		356 (85.0)	69 (90.8)	
Missing	20	8	1		27	2	
p27 Expression							
Negative	58 (18.4)	15 (10.1)	9 (27.3)	0.018	51 (12.1)	31 (40.8)	<0 001
Positive	258 (81.6)	134 (89.9)	24 (72.7)		371 (87.9)	45 (59.2)	
Missing	18	7	1		24	2	
Cyclin D1 expression							
Negative	63 (20.0)	23 (15.4)	10 (30.3)	0.129	90 (21.4)	5 (6.6)	0.003
Positive	252 (80.0)	126 (84.6)	23 (69.7)		331 (78.6)	71 (93.4)	
Missing	19	7	1		25	2	

^a^*n* (%); ^b^Kruskal-Wallis or Mann–Whitney U test. *MSI* microsatellite unstable, *MSS* microsatellite stable. One case with mutual codons 12 and 13 mutation was excluded from the analyses related to KRAS mutational status.

BRAF mutation was significantly associated with older age, female sex, proximal tumour location, low differentiation grade, mucinous tumour type, MSI and expression of cyclin D1, and inversely associated with beta-catenin overexpression, p53 positivity and p27 expression.

### Prognostic significance of KRAS and BRAF mutations

Hazard ratios for CSS according to KRAS and BRAF mutation status in the entire cohort, and strata according to sex, are shown in Table 3. In the entire cohort, a similar survival was seen for patients with KRAS wild-type and codon 12-mutated tumours, while patients with tumours harbouring a KRAS codon 13 mutation had a significantly reduced CSS (HR = 1.94; 95% CI = 1.18–3.19) in unadjusted, but not in adjusted analysis. KRAS codon 13, but not codon 12, mutation was also significantly associated with poor prognosis in women (HR = 2.58; 95% CI = 1.31–5.09) in unadjusted, but not in adjusted analysis. The KRAS mutation status was not prognostic in men.

**Table 3:** Risk of death from colorectal cancer according to KRAS codons 12 and 13, and BRAF mutations

	**Entire cohort**		**Women**		**Men**	
	**HR (95% CI)**	***n *(events)**	**HR (95% CI)**	***n *(events)**	**HR (95% CI)**	***n *(events)**
KRAS status (unadjusted)						
Wild-type	1.00	334 (113)	1.00	177 (52)	1.00	157 (61)
Codon 12-mutated	1.05 (0.76–1.45)	156 (54)	1.32 (0.85–2.05)	83 (32)	0.81 (0.50–1.32)	73 (22)
Codon 13-mutated	1.94 (1.18–3.19)	34 (18)	2.58 (1.31–5.09)	18 (10)	1.42 (0.68–2.96)	16 (8)
KRAS status (adjusted)						
Wild-type	1.00	273 (87)	1.00	146 (39)	1.00	127 (65)
Codon 12-mutated	1.02 (0.69–1.51)	132 (44)	1.29 (0.75–2.22)	67 (24)	0.74 (0.42–1.30)	65 (20)
Codon 13-mutated	1.37 (0.74–2.54)	28 (13)	1.83 (0.79–4.23)	15 (7)	0.87 (0.34–2.72)	13 (6)
BRAF status (unadjusted)						
Wild-type	1.00	446 (154)	1.00	227 (75)	1.00	219 (79)
Mutated	1.32 (0.90–1.94)	78 (32)	1.23 (0.74–2.04)	50 (19)	1.56 (0.87–2.81)	28 (13)
BRAF status (adjusted)						
Wild-type	1.00	370 (122)	1.00	184 (56)	1.00	186 (66)
Mutated	1.56 (0.87–2.79)	63 (22)	1.47 ( 0.65–3.33)	44 (14)	3.50 (1.41–8.70)	19 (8)

Adjusted analysis included age (continuous), sex, T stage (I-II, III, IV), N stage (0, 1, 2), M stage (0, 1), differentiation grade (high-intermediate vs. low and vascular invasion (+/−/unknown)), KRAS mutation status (wild-type, codon 12 mutation, codon 13 mutation) and BRAF mutation status (wild-type, mutated). One case with mutual KRAS codons 12 and 13 mutation was excluded from the analyses related to KRAS mutational status.

There were no significant associations of BRAF mutation with CSS in the entire cohort or in women, neither in unadjusted nor in adjusted analysis. In men, BRAF mutation was not prognostic in unadjusted, but in adjusted analysis (HR = 3.50; 95% CI = 1.41–8.70). This finding led us to investigate whether the prognostic value of BRAF differs in different disease stages in men and women and found that BRAF status was particularly prognostic in lymph node-positive disease in men, but not in women (data not shown).

Specific point mutations in KRAS codon 12 or 13 had no significant impact on survival, neither in the entire cohort nor in strata according to gender (data not shown). Similar results were observed for the overall survival (data not shown). KRAS and BRAF mutation status did not predict response to standard adjuvant chemotherapy in curatively treated patients with stages III and IV disease (data not shown).

### Prognostic value of BRAF mutation according to MSI status

As BRAF mutation has been previously reported to be associated with a particularly poor survival in cases with microsatellite stable (MSS) tumours [8,15,30,31], we also examined whether the prognostic value of BRAF mutation differs by MSI status, overall and stratified for sex. As shown in Table 4, BRAF mutation was overall associated with a significantly shorter CSS in patients with MSS tumours in unadjusted analysis (HR = 2.36; 95% CI = 1.44–3.86) and borderline significant in adjusted analysis (HR = 1.80; 95% CI = 0.98–3.28). BRAF mutation was not prognostic in MSI tumours. Again, no prognostic significance was found for BRAF mutation in women, either in MSS or in MSI tumours. In men, BRAF mutation was an independent factor of poor prognosis in MSS tumours (unadjusted HR = 3.46, 95% CI = 1.78–6.74; adjusted HR = 4.91, 95% CI = 1.99–12.12). Adjusted analysis was not performed in MSI tumours due to the small subgroups.

**Table 4:** Risk of death from colorectal cancer according to BRAF mutation and by microsatellite instability status

	**Entire cohort**		**Women**		**Men**	
	**HR (95% CI)**	***n *(events)**	**HR (95% CI)**	***n *(events)**	**HR (95% CI)**	***n *(events)**
BRAF status-MSS tumours (unadjusted)						
Wild-type	1.00	384 (135)	1.00	194 (64)	1.00	190 (71)
Mutated	2.36 (1.44–3.86)	29 (18)	1.73 (0.83–3.61)	16 (8)	3.46 (1.78–6.74)	13 (10)
BRAF status-MSI tumours (unadjusted)						
Wild-type	1.00	28 (4)	1.00	16 (2)	1.00	12 (2)
Mutated	1.68 (0.53–5.36)	43 (10)	2.15 (0.46–10.12)	31 (8)	1.03 (0.14–7.31)	12 (2)
BRAF status-MSS tumours (adjusted)						
Wild-type	1.00	343 (118)	1.00	169 (54)	1.00	174 (64)
Mutated	1.80 (0.98–3.28)	23 (14)	1.37 (0.56–3.35)	14 (7)	4.91 (1.99–12.12)	9 (7)
BRAF status-MSI tumours (adjusted)						
Wild-type	1.00	27 (4)	-	-	-	-
Mutated	3.24 (0.39–26.92)	40 (8)	-	-	-	-

Adjusted analysis included age (continuous), sex, T stage (I-II, III, IV), N stage (0,1,2), M stage (0, 1), differentiation grade (high-intermediate vs. low and vascular invasion (+/−/unknown)) and KRAS mutation status (wild-type, codon 12 mutation, codon 13 mutation). One case with mutual KRAS codons 12 and 13 mutation was excluded from the adjusted analysis.

## Discussion

In this study, we have investigated the prognostic significance of KRAS codons 12 and 13, and BRAF mutations in incident colorectal cancer from a large prospective cohort study, with particular reference to sex-related differences. As regards to the KRAS mutation status, the results demonstrated a significant association of KRAS codon 13 mutation, but not codon 12, with poor prognosis, but this significance was not retained in adjusted analysis. These results support precious findings by Bazan et al. who reported KRAS codon 13 mutation to be an independent predictor of a poor prognosis [5]. Samowitz et al. have also described similar associations, but only borderline significant [13]. Other studies have further reported any KRAS mutation to be associated with poor outcome [6-8,32]. In the present study, subgroup analysis revealed that KRAS codon 13 mutation was only prognostic in women and not in men, but only in unadjusted analysis. While no significant associations were found between KRAS mutations and sex, the significant association of KRAS mutation with MSS tumours found here is in concordance with the results from previous studies [6,7,9]. Further, the associations of KRAS codon 13 mutation with metastatic disease and codon 12 mutation with mucinous tumour type have also been demonstrated previously [5]. Taken together, these findings further indicate that specific KRAS codon mutations have different impact on protein functionality and should be taken into consideration when evaluating KRAS mutation status in the clinical setting. Furthermore, in light of the accentuated prognostic impact of KRAS codon 13 mutation in women, it will also be of interest to perform further studies on the associations of hormonal factors with KRAS mutation status in CRC.

In analysis of the entire cohort, BRAF mutation was not prognostic in women, but in men; BRAF mutation was significantly associated with an impaired survival in adjusted, but not in unadjusted analysis. This may be explained by the fact that the prognostic impact of BRAF mutation status was stronger in, e.g. lymph node-positive disease in men, but not in women. It is well established that BRAF mutation, in contrast to KRAS mutation, is associated with MSI [9,10,16,33] and female sex [10,33,34], and our findings further validate this. In MSS tumours, BRAF mutation was significantly associated with a reduced CSS in unadjusted analysis, and was borderline significant in adjusted analysis. These findings are in concordance with several previous studies [9,15,17,35], indicating that BRAF-mutated/MSS tumours represent a more aggressive tumour phenotype. However, the results from this study further demonstrate that BRAF-mutated/MSS tumours were not significantly associated with poor prognosis in women, but an independent predictor of a reduced CSS in men.

To date, no biomarkers have yet been incorporated into clinical protocols for prognostication and treatment stratification of CRC patients in the adjuvant setting, which still relies entirely on the assessment of conventional clinicopathological factors and patient performance. Approximately 20% of patients with stage II disease will develop recurrent disease and although several risk factors, e.g. <12 examined lymph nodes, T4 disease, vascular or neural invasion, low differentiation, acute operation and tumour perforation, have been suggested, the benefit from adjuvant chemotherapy in this patient category is rather modest [36,37]. Our results further indicate that this algorithm is not only in need of additional molecular biomarkers, but that sex should also be included as a variable.

The main purpose of this study was to analyse and compare the prognostic significance of KRAS and BRAF mutations in women and men, irrespective of adjuvant treatment. However, potential differences in response to standard adjuvant chemotherapy in curatively treated patients with stages III and IV disease according to KRAS and BRAF mutational status, MSI status and sex were also examined, whereby no significant differences were found. Therefore, the finding of BRAF mutation being a particularly negative prognostic factor in men warrants validation in additional independent patient cohorts, which may well be done in the retrospective setting, before further prospective study.

Although the proportion of patients in this study that may have received EGFR inhibitors upon recurrent disease is likely to be negligible, it is also important to consider potential sex differences when evaluating the results from trials related to response to EGFR inhibitors. For instance, results from several trials have demonstrated a significantly better response to EGFR tyrosine kinase inhibitors in women with advanced non-small cell lung cancer compared to men [38,39].

The higher prevalence of BRAF-mutated tumours in women, together with the lack of prognostic impact of BRAF mutations in female CRC, indicates the possibility of a link between hormonal factors and BRAF mutation status in CRC. Therefore, an influence of anthropometric and lifestyle factors is also plausible [22] and should be pursued in future studies.

As a cautionary remark, several of the here presented results, in particular related to gender, are derived from analyses of rather small subgroups and need validation in additional patient cohorts. The validity of the findings are however strengthened by the expected associations of KRAS and BRAF mutations with clinicopathological factors, e.g. KRAS and BRAF mutations being mutually exclusive [20], the significant associations between BRAF mutation, MSI [15,20,40] and mucinous phenotype [41,42].

Apart from established clinicopathological parameters, we have also examined associations of KRAS and BRAF mutation status with several other investigative factors, i.e. beta-catenin overexpression and expression of p53, p21, p27 and cyclin D1. The observed inverse association between BRAF mutation and beta-catenin overexpression has been described earlier [43,44] and is also in line with the previous findings of beta-catenin overexpression being associated with good prognosis in this cohort [28]. The observed associations between BRAF mutation and expression of p21 and cyclin D1, and loss of p27 and p53 expression have also been previously reported [45-48].

The Malmö Diet and Cancer Study is a population-based cohort study, wherein a potential selection bias compared with the general population must be taken into consideration. As previously denoted [23], the frequency of emergency surgery was only 8.3% which is lower than the commonly reported frequency of approximately 25% [49,50], which may reflect a higher awareness of CRC among study participants. On the other hand, the distribution of clinical stages at diagnosis is in line with the expected [23,25].

## Conclusions

In conclusion, the results from this large prospective cohort study provide further support to the accumulating evidence of BRAF-mutated microsatellite stable colorectal cancer having a particularly impaired prognosis. The finding of BRAF mutation being an independent factor of poor prognosis in male, but not in female colorectal cancer, both overall and in MSS tumours, is however novel and merits further study. Moreover, the findings in this study further emphasize the importance of taking sex into consideration in all cancer biomarker studies, since this may enable the development of more accurate prognostic nomograms for identification of patients with high-risk disease.

## Competing interests

The authors declare no competing interests.

## Authors’ contributions

SW carried out the statistical analyses and drafted the manuscript. BN, NZ and MS carried out the pyrosequencing analyses and helped draft the manuscript. KEL carried out some of the immunohistochemical analyses. SW and JE collected the clinical data. JE and KJ conceived of the study, participated in its design and coordination and helped to draft the manuscript. All authors read and approved the final manuscript.
